# Global transformation of disaster sciences into risk reduction education system in Indonesia

**DOI:** 10.4102/jamba.v14i1.1257

**Published:** 2022-11-30

**Authors:** M. Yakub Aiyub Kadir, Nurmalahayati Nurdin

**Affiliations:** 1 Department of International Law, Faculty of Law, Syiah Kuala University, Banda Aceh, Indonesia; 2 Department of Chemistry, Faculty of Education, State Islamic University Ar-Raniry, Banda Aceh, Indonesia

**Keywords:** Disaster risk education, global education, disaster science, United Nations, Indonesia

## Abstract

This article explores the origin of disaster risk reduction (DRR) knowledge in the global context and how it evolved in the education system of Indonesia since its independence in 1945 up to 2018. It argues that DRR education in Indonesia is a marriage of global knowledge and local experiences, which lacks preparedness (knowledge and skills) and has resulted in humanitarian crises. Using a historical approach, this article found that the transformation of global knowledge and DRR education in Indonesia has not yet been effectively implemented, while on the ground, there has been an increasing development of community programmes on DRR education.

**Contribution:**

The contextualisation of global and DRR education in Indonesia, in terms of policy and institutional networks, can identify the interrelated elements of DRR education, that is, disaster science and pedagogic and human resource development. These need to be evaluated for better formulation of policy in the future.

## Introduction

This article describes a theoretical understanding of disaster risk reduction (DRR) in the global context and how it evolved in the Indonesian education context and in a contrary. The emergence of global education on DRR was an integral part of the response of states over the globalisation era and its associated natures. It is a relevant concern in the current domain on preparing the future generation with new knowledge, experiences and skills on various global issues, which was not much of a concern before. In this context, education is a life experience and the development process to obtain knowledge and skills, which become instruments of understanding and dealing effectively with future hazard and vulnerability situations.

This article covers the response of the United Nations (UN) over the lack of disaster knowledge and preparedness in developing countries which led to the formulation of the Yokohama Strategy in 1994. It argues that the patterns and meanings of DRR in Indonesia have been radically altered since the Aceh tsunami of December 2004, which globally triggered the Hyogo Framework for Actions (HFA) agreement in 2005. Coincidently, it found momentum under the Reformation era, 1998–2018, which is being re-theorised as global DRR as well as national DRR in Indonesia.

## Methodology

This article utilises historical approaches to link up the global development of disaster science into disaster education and how it evolved within the Indonesian education system, that is, how the DRR knowledge and its conceptual changes transform from local, national and global perspectives and the interlink between them. Many resources from the United Nations documents and from the Indonesian education development were traced and analysed in terms of developing a connection between DRR knowledge, scientific development and various disasters, hazards and vulnerabilities, with reference to Indonesia.

## Findings and discussion

### The origin of disaster risk reduction education

#### Sporadic response to global disaster

Despite disasters occurring for centuries, there is limited literature describing DRR education prior to the establishment of the UN in 1945. The DRR initiative was earlier related to humanitarian relief for refugees post–World War II. It then formed humanitarian relief in cases of disasters upon the occurrence of specific events calling for emergency actions. In the early days when large disasters occurred, the Social and Economic Council and the General Assembly invited the World Health Organization (WHO), World Food Program (WFP) and the United Nations Children’s Fund to assist people affected by disasters. There was grave concern regarding emergency situations post disasters, and in some regions it involved rehabilitation and reconstruction, such as the UN General Assembly (UNGA) response to the powerful earthquake in the Yugoslavian city of Skopje in 1963, a hurricane in the Caribbean and 2 years of severe drought in Afghanistan (see the UNGA Resolution 2757 [XXVI] 1971).^1^

Hence, between 1945 and 1965, DRR was more of a sporadic short-term response to areas in countries that had been affected by disaster. In the following years, the UN initiated a more systemic approach at the request of the Social and Economic Council Resolution 1049 (XXXVII) 1964 to the General Assembly to study the types of assistance. This was thereafter consolidated, reviewed and recommended under the UNGA Resolution 2034 (XX) 1965 on the UN’s role in humanitarian relief programmes, including the position of the UN Secretary-General as the coordinator, to rehabilitation and reconstruction, in addition to funding allocation. The resolution places equal emphasis on the importance of promoting understanding among governments with regards to the necessity for ‘predisaster planning’ and the establishment of a single national responsible authority for disaster management.

It was the first resolution to consider the term ‘predisaster planning’, later extensively known as ‘disaster preparedness’, in which the UN could offer advice and assistance upon requests from governments regarding all types of planning measures. Despite the lack of response in many developing countries, it increasingly attracted global attention. In a report published in 1970, the Secretary-General emphasised the importance of national responsibility over predisaster planning (Macalister-Smith 1985).

The UNGA Resolution 2435 (1968) on Assistance in Case of Disaster stated that the extended assistance for developing countries and the need for ‘scientific research and modern technology in reducing the impact of disasters on man and society’ has been demanding. Points 1 and 2 of this Resolution highlighted to governments the need to establish administrative measures and scientific research on disaster risk, which was theorised as ‘hazards and vulnerability concept’, and to encourage preventive and protective measures, such as the construction of safe houses and the like. However, many developing countries lacked the expertise as they were newly independent countries and wanted to focus on recovering political and economic stability. Equally, the UN failed to give a clear diagnosis of the problem and provide clear and applicable recommendations for developing countries prone to disasters.

The repetitive rhetoric of the need for governments to consider preventive and scientific research on disaster risk and hazards within various UNGA Resolutions during the period 1968–2004 was rather unsuccessful. The Secretary-General’s review of the 2034 Resolution (1971) recognised the unsatisfactory progress of the Resolution and provided an expansion of the UN role from emergency relief to the new system of UN management, such as the designation of the UN Relief Coordinator and its office under the Secretary-General and emphasis on the afflicted countries’ role to increase capacity to prepare and respond to disasters.^2^ This has urged developing nations to consider their internal capacity to prepare for disasters.

The UN then developed a more collective effort to support the international community in responding to several disasters in developing countries. The UN has played a more effective role in post-disaster response, supported by its affiliated agencies, such as the United Nations Development Program (UNDP). It called for the Secretary-General to appoint a Disaster Relief Coordinator to mobilise and coordinate relief activities in the field. For example, point (f) of this Resolution 2816 (1971) highlighted the need ‘to promote the study, prevention, control, and prediction of disasters, including the collection and dissemination of information concerning technological developments’. This point was changed to promote DRR education, as part of the duty of the UN Disaster Relief Coordinator (UNGA Resolution 2816).

#### The globalisation of disaster risk reduction education

The purpose of the declaration on the International Decade for Natural Disaster Reduction (IDNDR) in 1989 was to raise global concern on coping with disasters around the world, particularly in developing countries. It raised concerns on environmental protection for prevention and mitigation of disasters, ‘recognizing the importance of environmental protection for the prevention and mitigation of natural disasters’^3^ (UNGA Resolution A/RES/44/236 1989). It also pointed out ‘… that the international community as a whole has now improved its capacity to confront this problem’. The term ‘disaster reduction’ began to be used in this resolution, as implicitly produced mitigation and preventive measures to encourage governments to develop national policies on disaster reduction systems. Therefore, the effort and the capacity to mitigate disaster risks remains at a low level. Since this declaration in 1989, the UN has taken a more active role in supporting developing countries in disaster preparedness and mitigation. However, the idea was not very clear in regard to what form the DRR would apply. The organs, organisations and bodies of the UN system are urged to accord priority, as appropriate and in a concerted manner, to disaster preparedness, prevention, relief and short-term recovery, including economic damage risk assessment, in their operational activities. The Secretary-General was requested, in this regard, to ensure that adequate means are made available to the Office of the United Nations Disaster Relief Coordinator so that it may diligently discharge its specific role and responsibilities in the field of disaster mitigation (Annex UNGA Resolution A/RES/44/236 1989, point 5).

In 1989, the UN explicitly manifested and managed the DRR programme in developing countries as the establishment of the secretariat at the UN Office in Geneva, in close association with the Office of the UN Disaster Relief Coordinator, with its members drawn from the international community of disaster reduction experts and other relevant experts. The secretariat was responsible for the day-to-day coordination of Decade activities and provided substantive and secretarial support to the special high-level council and the scientific and technical committee, as well as for other related activities (Annex UNGA Res 1989, point 14 [a, b]). The UN then established:

A scientific and technical committee on the International Decade for Natural Disaster Reduction (IDNDR), consisting of twenty to twenty-five scientific and technical experts selected in consultation with their Governments on the basis of their personal capacities and qualifications, including experts from the organs, organizations and bodies of the United Nations system. (Annex UNGA Res 1989, point 12)

This technical committee was considered the first think tank in the context of developing DRR in education at a global level.

Additionally, the establishment of the Yokohama framework in 1994 is important, as is the immediately following Kobe earthquake in 1995. These two events contributed to the global development of DRR, to raise a collective awareness for the essential of DRR, in particular for developing countries which are ‘… least equipped to cope with them’ (World Conference on Natural Disaster Reduction, Yokohama, Japan, 23–27 May 1994).

Then in December 2002, the UNGA Resolutions 57/254 declared the ‘Education Decade’ for sustainable development 2005–2014, under the United Nations Educational, Scientific and Cultural Organization (UNESCO). In this resolution, education for disaster mitigation became more explicit. It was then that DRR education became a global project in developing countries that are mostly affected by disasters.

#### Environmental and sustainable development

Soon after, the various UNGA Resolutions in response to a disaster in El Salvador and the accumulation of the lack of preparedness for disaster risks in developing countries encouraged the UN to develop an International Strategy for Disaster Reduction under the UNGA Resolution A/RES/56/195 (2002). In point 24, the resolution requests the Secretary-General to submit to the General Assembly a report on the implementation of the present resolution, including criteria and modalities for the selection of the nonpermanent members of the Task Force and on the progress made in the implementation of the International Strategy for Disaster Reduction, under the item entitled ‘Environment and Sustainable Development’ (UNGA Resolution A/RES/56/195 21 January 2002).

This resolution can be considered the first initiation to invite all governments and relevant organisations of the UN system to strengthen national participation, in particular of disaster-prone countries, in order to achieve the Sustainable Development Goals (SDGs) and objectives with the full utilisation of scientific and technical knowledge and strengthening of global and regional approaches. It provided 24 detailed policies for DRR education development, including reaffirming the interagency task force for DRR, a national focal point, to encourage adequate financial and administrative resources for the effective functioning of the Task Force and the interagency secretariat in implementing the strategy to establish national platforms and so forth (UNGA Resolution A/RES/56/195, 21 January 2002). In addition, some activities were also conducted to disseminate disaster information through all available channels, including handbooks and information systems. The information is necessary for the effective management of international cooperation in the fields of disaster prevention, early warning, response, mitigation, rehabilitation and reconstruction and as a vehicle to promote a global culture of DRR (UNGA Resolution A/RES/56/195 2002; UNGA Resolution A/RES/55/240 2001).

The emergence of global knowledge pertaining to DRR education was relatively new, embedded in the environmental and sustainable development issues, until the emergence of HFA 2005, Building the Resilience of Nations and Communities to Disasters, in which DRR become an independent field in the global sphere. But there was a huge change when the tsunami hit Aceh province in 2004. The disaster not only compelled the Indonesian government but also the UN to consider schools as places that are vulnerable to disaster risks. Since then, there has been enormous progress at both the national and international levels focusing on DRR education to strengthen the ability of schools and communities to manage disaster risk. This is evident in the global campaign that was conducted under the theme of ‘DRR begins at Schools’ that took place at the UNESCO headquarters in Paris on the International Day for Disaster Risk Reduction (IDDRR) on 11 October 2006. The campaign explored two issues of DRR education: the integrating of DRR-related subjects in the school curriculum and school safety in terms of safe standards for school construction. This campaign managed to raise awareness in numerous countries, including Indonesia. Hence, the disaster triggered the accumulation of global and national knowledge regarding DRR education (Djalante & Garschagen 2017).

The emphasis on environmental hazards is historical in nature, as the issue of environmental hazards becomes a part of the mainstream in development of DRR education in the global sense. In this article, the environmental hazard has two directions: to show how the environmentalism movement has triggered DRR knowledge and how environmental hazards led to DRR’s reduction of vulnerabilities. The latter is a wide area which needs to be discussed further.

The development of DRR education, also identified by the term ‘hazard’, is the probability of occurrence of a potentially damaging phenomenon, and vulnerability describes the degree of loss resulting from the occurrence of the phenomenon. Both terms are strongly interrelated and lead to a more sophisticated nature of DRR knowledge. Every country has its own hazards and vulnerabilities, following the nature of their disasters and their preparation to prevent and respond to such disasters. Disaster risk reduction education approaches vulnerability reduction, including implementing building codes; insurance and social protection (risk); emphasising economic diversity and resilient livelihoods; knowledge and awareness raising; and preparedness measures. In this development, more research is required to interpret such concepts in a more practical and pedagogic manner. Local knowledge accumulation has, however, been developed under ‘DesInventar Sendai’ system software (UNISDR, Geneva, Switzerland) to help more than 90 countries worldwide to develop sustainable disaster information management systems, aligned with the Sendai Framework monitoring process. The system allows one to analyse disaster trends and impacts through a large range of sectoral and socio-economical dimensions.

It is significant for DRR education to formulate materials and pedagogic matter for raising such awareness (GAR 2015).The focus of DRR needs to move ‘from managing disasters to managing risks, if it is to contribute to making development sustainable’ (Global assessment report on disaster risk 2015). Thus, the Global Assessment Report on Disaster Risk Reduction (GAR) 2015 has pointed out the urgency of DRR education in a rhetorical manner, and further research would be needed to develop a curriculum and teaching methodology for managing hazards for reducing social vulnerability. It also emphasises the close link between disaster risk and sustainable development and explores prospective, corrective and compensatory risk management approaches as a way to integrate it in development activities, in order to avoid risk generation and accumulation, a move from managing disasters to managing risks (GAR 2015).

In the following years, DRR education has become embedded in major global frameworks, for instance, the Sendai Framework for Disaster Risk Reduction (SFDRR), SDGs, the Paris Agreement on Change, the Addis Ababa Action Agenda on Financing for Development (AAAA) and the New Urban Agenda in 2016. The various global frameworks for disaster management indicated the complexity of the development of disaster resilience thinking and how it impacts the postcolonial fragility of governance and institutional networks (Chandler 2014).

#### Global education and disaster risk reduction

The emerging term ‘global education’ is essential to cover the emerging global notion and issue of DRR initiatives, which have not been well distributed and understood across the world, but it has an impact even on daily life (Standish 2012). It can be said that learning about global issues is a process of increasing self-awareness: for instance, what do students think about disasters, and what do they think should be done to reduce the risk of them occurring? The study of global issues does not just seek to elicit the thoughts of students regarding global issues but how they can actively involve themselves in finding the answers (Standish 2012). In fact, a new reason for education can be seen as developing the relevant knowledge to prepare students to deal with global issues in society (White 2007). It is also no longer just for the people who may become victims but for all the people.

Disaster risk reduction is an element of global issues because of the fact that it needs a solid effort from both the national and the international level to build an understanding and awareness on disaster risk, and therefore, education has an essential part and place in it. Disaster risk reduction education requires resilience thinking to mitigate disasters, the capacity to manage disaster risks through more viable resilience strategies (Chandler 2014).

The development of global education in Indonesia has been distinct from those in Western states, that is, the United States (US) and the United Kingdom (UK). Global education in Indonesia is more complex because it also has a colonial influence. It was expected to be a solution for current limitations in the education system or be an essential part of national education development. Integrating global and national experiences of DRR has contributed to the development of DRR initiatives, in the form of policy and institutional networks.

The turning point of the Indian Ocean disaster created a fundamental change in the way disaster risks and disaster impacts have been viewed in Indonesia and globally. The formulation of the five priorities of HFA in 2005, covering (DRR governance; risk assessment and early warning; knowledge and education; reducing the underlying risk factors; and disaster preparedness and response) has created a significant development of DRR education. The HFA provides a monitoring and scale review to measure the level of progress made by each country. In general, Indonesia has moved gradually from a score of 3.0 (not substantial progress) to 3.7 (nearly substantial progress)^4^ during the period 2013–2015 (BNPB 2015b). Education has therefore become the core of the HFA, as its role may directly or indirectly cover the five actions proposed. Education in schools has its role in preparing future generations in sustainable development. Nonetheless, mainstreaming DRR in the existing school system demands a large paradigm shift in Indonesia, as it is a part of shifting the paradigm from the traditional system to the modern school system, including the curriculum.

The concept of interdependence (Garlake 2007) as an essential component of DRR education has strong links to the nature of DRR in global education. A disaster has no direct relationship with state boundaries; it may occur in one and more states simultaneously, or the same disaster may happen in many, and the impact will also go beyond state boundaries, even covering the entire world, such as climate change issues. This reality has obviously globalised DRR education to develop the knowledge and awareness of students. By definition, global DRR education refers to the knowledge, values and skills that are orientated towards issues that are global in nature. In science, students are taught about patterns of disasters, the environment and so forth. However, in the postmodern world, the meaning of DRR itself has changed, and not just in scale. Thus, global education and world studies run counter to the idea that education is a means of inducting children into knowledge and culture through a subject-based curriculum. Instead, global education means studying real-world problems, engaging with them and learning to see issues from the perspective of others (Standish 2012:20).

### The transformation of global education into disaster risk reduction in Indonesia

The ethical policy has been a turning point for colonised people of the East Indies (currently Indonesia) to interact with global knowledge, so as to shift from marginalised and stagnant religious-based education to broader knowledge, despite the extremely limited elites of *Pribumi* [indigenous Indonesian] and the limited territory in Java Island (see Suminto 1984). During colonialism, education was limited to *Pribumi* elites on Java Island. The situation meant the political power was dominant on Java because more people were educated. However, the situation changed after the Reformation movement in 1998. The increasing demand for decentralisation has contributed to the increasing local nationalism referring to their historical existence. This has become a dilemma for Indonesia in redesigning global education under a decentralised system. In the meantime, the 21st century has also given Indonesia the opportunity to compete with other states, but thus far the country has made little progress, as it remains under development and obscure in terms of its nature and characters. Hence, Indonesia has opened some spaces for external knowledge which directly or indirectly have an impact on people and the state. In the initial stage, there is a transformation regarding understanding global education, from the spirit of anticolonialism to the common interest of all states facing common global challenges, such as DRR issues (Somantrie 2010). The transformation came through several notions, as detailed next.

#### The concern on human rights (children’s rights)

The Reformation era post Suharto (1998 onward) was outstanding as it opened a new paradigm for Indonesia to adopt human rights, in particular, the rights of children in a disaster emergency under the term ‘Special Service Education’ unit in the Ministry of Education. The government has to ensure schoolchildren have access to education post disaster. Although no DRR knowledge and skills were explicitly formulated, the consciousness of disaster risks had the potential to accept DRR education later on.

During the Suharto regime, Indonesia was considered a restricted country regarding global issues. All aspects were centrally controlled and monitored by the military. Such circumstances hindered the government from being open with the public and to global changes. The concern was to develop a new nationalistic Indonesia, united in politics, society and culture. During the Reformation Order, President B.J. Habibie (1998–2000), a scientist who had graduated in Germany, made use of his knowledge and experience to promote democracy and transformed several global norms into the Indonesian system.

The global human rights issue was the first concern of President Habibie, who introduced the global human rights agenda into the Indonesian system by establishing the *Human Rights Law 1999* and *Human Rights Court Law 2000* (Kadir 2009). It was the first time Indonesia had adopted the global issue of human rights, in contrast to the previous autocratic and militaristic government of Suharto. From these general human rights issues, the rights of children in disadvantaged situations and areas gained momentum, and *Law 23/2002* on child protection was established. It covered rights for children in disadvantaged areas and indigenous communities, among the economically disadvantaged and those affected by disasters. Then in 2003, remarkably, the word ‘disaster’ was inserted into *Law 20/2003* relating to the National Education System. It can be said that at that stage, national education was becoming more concerned about the issue of national disasters. The development of the children’s rights section under human rights law is considered a part of the global transformation so that children can get basic access to their needs and be protected during and after a disaster. The limited rights of children in disaster areas was then extended in *Law 24/2007*, which revealed the primary concern of every person to receive an education, training and advice and skills in the manifestation of disaster management, both in nondisaster and disaster situations. Consequently, human rights have become the underlying foundation of DRR management and education in Indonesia. The Indonesian government has explicitly recognised that DRR education is the right of every citizen and that the government has an obligation to fulfil these rights under *Law 24/2007* and also previously in special service education in *National Education Law 2003*.

Fulfilling the rights and concerns of children during disaster situations became the underpinning issue of the establishment of safe schools as safe places for children facing disasters. Since 1998, Indonesia has ratified the UN Convention on the Rights of the Child. It strongly relates to assuring that children can be saved from disaster risk and the subsequent impact. In *Law 23/2002* on child protection, the rights of children became more important (Sukemi & Andriono 2014). Despite the term ‘the right of every person’ described in *Law 24/2007*, in most of the implementation, the term ‘the right of children’ is dominantly applied, as in DRR modules, where the aim of the module was ‘to protect the rights of children’ in terms of survival and quality sustainable basic education in DRR (Ministry of Education & UNICEF 2015). The term ‘rights’, noted to be an influence by global human rights movements, has politically and economically transformed Indonesia since the Reformation era in 1998, which is to say that the rights-based approach of DRR education was adopted in the formulation of *Law 24/2007*. The expression of DRR education manifested through the rights-based approach in Indonesian legislation, transformed from global knowledge on DRR in HFA 2005. In other words, despite the fact that local disasters require DRR education, the limited expression in local and national knowledge has encouraged Indonesia to adopt a global language.

In many policies, regulations and DRR-related material in Indonesia, the consideration was the protection of the rights of children to achieve a quality and sustainable education. On this basis, DRR education, including Sekolah Siaga Bencana (SSB) was developed (Ministry of Education & UNICEF 2015). As a UN-associated agency concerned with education, UNESCO has played a pivotal role in developing DRR education that has been incorporated into the Indonesian system. The Japan International Cooperation Agency (JICA) has also continuously supported Indonesia to transfer the lessons learned from Japan to Indonesia (Ikeda & Mulyadi 2012).

However, the Aceh tsunami in 2004 raised more awareness, which made it more explicit under the commitment of 168 states with regards to HFA 2005–2015. This then, remarkably, changed the paradigm of DRR in Indonesia by developing a national legal framework of *Law 24/2007* on disaster management. Subsequently, the National Education Ministry issued a Circular Letter to mainstream DRR education in schools, followed by Perka BNPB Number 4/2012 on the Guideline for the Implementation of Safe Schools, in addition to the mainstreaming of children’s rights adopted initially by Lembaga Ilmu Pengetahuan Indonesia/Indonesia Institute of Science (LIPI) under the Children Science Support (CSS) programme in some areas in 2006 (Rafliana 2017). It is important to mention that this global knowledge was essentially intended for political and economic reasons rather than for education purposes. Nonetheless, it has contributed to a transformation of DRR from merely political-economical purposes into the education aspect of disaster, subsequently known as DRR education.

#### Environmental (climate change) and sustainable development

Disaster risk reduction issues can also be traced from the element of Education for Sustainable Development and Environmental Education in the late Sixties. Since then, the emphases on environmental education has shifted from conservation of the countryside in the Sixties and Seventies (plants, trees, hedgerows, wildlife), to national and global problems in the Seventies and Eighties related to pollution, resource depletion and global warming, to issues of sustainability in the 21st century. This development was greatly influenced by the UN-associated institution, that is, UNESCO, in formulating the idea to be more feasible and understandable (Palmer 1998). It indicated that DRR was a growing concern for current and future education, as Toffler (1974) noted that:

All education springs from images of the future and all education creates images of the future. Thus, all education whether so intended or not, is a preparation for the future. (p. xxii)

The influence of global forces on DRR increasingly appeared. In 2015, the module developed by the Ministry of Education and UNICEF (2015) set out explicitly that:

Mitigation disaster risk as a long-term project, part of sustainable development, through knowledge, innovation, and knowledge of safety and resilience culture in all education units, as referred to in HFA, which Indonesia is fully committed to. (p. 8)

To demonstrate its international commitment to DRR education, the commitment was reconfirmed in the Sendai Framework for DRR 2015–2030 (Ministry of Education & UNICEF 2015).

Current DRR in global discourse was believed to be part of environmental learning to develop the environmental awareness of the population to be a sustainable society. Disaster risk reduction as an emerging issue in disaster-vulnerable countries may become an independent global issue and discipline, as a common goal and interest of global citizens. However, this movement was created by the ‘developed world’ via the Organization for Economic Co-Operation and Development (OECD) in the Nineties (OECD 1995:3). The unexpected high number of victims and destruction has attracted 168 states, including Indonesia to create a global commitment for disaster risk mitigation, under what is known as ‘the HFA’ in 2005.

Following this fact, the Indonesian government set up national action for DRR as Priority 5 (RAN PB) 2010–2012, which translated from Priority 5 of the HFA 2005–2015. It was said ‘to strengthen disaster preparedness and respond effectively to all levels of the community’. In particular, in Priority 3 of the HFA 2005–2015, DRR education was shown as ‘using knowledge, innovation and education to establish a safe and resilient culture at all levels’ (Ikeda & Mulyadi 2012:11). Thus, there would be a need to develop a safe and resilient culture in school communities, including preparedness and the mitigation aspect.

#### The global ‘safe schools’ movement

School plays an important role in Indonesian society and is seen as a place to transfer information, knowledge and skills to the surrounding community. Consequently, school is an effective, dynamic and sustainable strategy in transforming disaster education (CDE 2011). In global development, there was a global campaign of the United Nations Office for Disaster Risk Reduction (UNDRR) in 2010–2011 focusing on the development of urban areas in terms of the theme ‘One Million Safer Schools and Hospitals Campaign’ to promote DRR in schools and hospitals. In May 2013 in Geneva, a significant communiqué from the Fourth Session of the Global Platform for DRR requested that:

A global safe schools and safe health infrastructures campaign be initiated in disaster prone areas with voluntary funding and commitments to be announced at the World Conference for Disaster Risk Reduction in 2015. (UNISDR 2015b)^5^

In 2015, the Third UN World Conference on DRR in Sendai, Japan, welcomed high-level government representatives and international organisations and announced more commitments to the Worldwide Initiative for Safe Schools (WISS) and school safety to be implemented globally. Under the motto ‘As of 2016, every new school will be safe from disasters’, the meeting highlighted the importance of building partnerships and using a holistic approach to promote safe schools as a global project. A set of tools were developed by the United Nations International Strategy for Disaster Reduction (UNISDR) to support governments in developing a holistic approach to school safety (UNISDR 2015a).

To support this spirit, the UNISDR has coordinated with WISS as an umbrella for the safe school global partnership programme that encompasses key safe school initiatives in supporting resilience in the education sector. The WISS work is focusing on developing DRR national strategies and the implementation of safe schools. The initiative covers safe learning facilities, school disaster management and DRR and Resilience Education. The WISS was endorsed by Global Alliance for DRR and Resilience in the Education Sector (GAD3RES) members and resulted in the political commitment of 21 ‘Safe School Leader’ countries to implement school safety on the ground (UNISDR 2015b). The Initiative also promotes good practices and the possibility for replication in, and offers technical supports for, other countries. This commitment shows that the education sector has become one of the main concerns of the UN for the coming years. However, this commitment has not yet been supported by innovative research on how to transfer and develop the pedagogic nature of DRR education.

This major framework has transformed Indonesia, including the institutional integration of DRR and the activities to address and reduce susceptibility to climate risks at school and local community level (Djalante et al. 2012). This transformation has come through institutional interaction and science knowledge development between experts from Western countries and Indonesian science institutions, besides the Ministry of Education. Finally, the term ‘safe school’ was subsequently added in the UNGA Resolution, then adopted in the Indonesian language as ‘Sekolah Siaga Bencana (SSB)’ or ‘Sekolah Aman’.

#### Natural science development

Essentially the essence of DRR education is the science of disaster in relation to natural science, that is, chemistry, physics, geology and Earth sciences and the like. Further development of this science would describe the nature of a disaster, its process and impacts. From this knowledge and understanding, humans (scientists) then develop knowledge on how to prepare and respond to disasters and the impacts. Moreover, DRR is considered new knowledge, and it has attracted more attention in recent decades. As science is far developed in Western countries, so DRR education has also flourished there and was then transferred to developing countries. Despite many disasters occurring in developing countries, the lack of science development has negatively contributed to the development of DRR science and education in these countries. It can be seen from several UNGA Resolutions cited above that the UN has continuously highlighted the importance of knowledge transfer relating to disaster preparedness.

Equally, the module developed by LIPI refers to the geological features of Indonesia as located on three continents. The knowledge of the causes and impact of a disaster, how to respond, how to mitigate the risks and the history of disasters in Indonesia (Krakatau volcanic eruption 1883, tsunami in Simelue 1907, Flores 1992, Aceh & Nias December 2004, Pangandaran July 2006) are essential with respect to DRR education (Hidayati et al. 2011; Rafliana 2017).

According to Rafliana (2017), the DRR concept was originally used in Western culture to solve social problems in society. It was a critique of the orthodox Christian churches that fuelled enlightenment and industrialisation, which then led to colonisation. Consequently, Indonesia essentially does not have any appropriate DRR knowledge; therefore, it will take time to embed foreign knowledge into the Indonesian system. While science in Indonesia remains very bureaucratic, it will be difficult to develop DRR in formal education. Rafliana (2017) highlighted the strong link of science development and DRR education in Indonesia which LIPI has been involved in. Rafliana (2017) pointed out that:

There were very few Indonesian scientists from the pre-independence era up to Soeharto (New Order) regime, who worked on disaster documentation, despite the numerous disaster events that occurred in the past (p. 411–441, 414).

During that time, disasters were not perceived as disruptive factors towards the government’s development agenda, which caused the absence of disaster knowledge during that time. The rationalisation of science during the period was not enough to improve the understanding of risk in society and prepare for future disasters. Moreover, the traditional knowledge embedded within communities was only vaguely recommending messages of preparedness concerning future threats (Rafliana 2017:414–415).

The establishment of disaster research in LIPI has predominantly focused on natural hazards, including geological dynamics, particularly tectonic plates, since 1963. Supported by Professor Kerry Sieh from the California Institute of Technology in the 1990s, LIPI’s researchers began their research on the Sumatran Subduction Megathrust in 1994 and Mentawai in 2002. During this time, LIPI researchers introduced knowledge on tsunamis and earthquakes, including some public preparedness by way of posters, public discussions and village meetings. This was one of the first attempts by LIPI to convey science to society. In Indonesia, nevertheless, the unprecedented Indian Ocean tsunami gave DRR momentum, not only in Indonesia but globally. It brought transformational changes in the way disasters are viewed and managed worldwide (Rafliana 2017).

#### Local catastrophic disaster 2004

Disaster risk reduction education became important in Indonesia after the local catastrophic disaster in December 2004, in response to the under-representation of DRR knowledge in the Indonesian system. It is important to consider the influence of the Aceh tsunami on DRR, since for many Indonesians this event shifted their social and political attitudes.

In reading the literature after the event, there seems to be two significant responses: a rise in concern over the lack of a DRR system in Indonesia and a call for more international participation to help those affected by the disaster. These two responses are not necessarily mutually exclusive; however, for some people this event enhanced their sense of humanity, while others wanted to break down barriers with other nations. Many writers and commentators have remarked on the prominence of national flags in streets, on cars, on houses and on clothing in the weeks after the tragic disaster. For many Indonesians, their reaction was one of national failure and desire to stand up for their country in opposition to this ‘new’ disaster. Moreover, Aceh had been closed from global and national media coverage as it was under martial law (1976–2005) (Kadir 2015). This period after the disaster demonstrates how quickly Aceh moved from being isolated to being recognised by the rest of Indonesia and the world.

In the first year of this rehabilitation of emergency response, international actors led the work in the field, as Aceh’s government had collapsed, and the central government of Indonesia also had no experience of responding to such a devastating disaster. The first institution was then developed as a coordinating body for the rehabilitation and reconstruction of Aceh-Nias (Badan Rehabilitasi dan Rekonstruksi Aceh-Nias [Agency for the Rehabilitation and Reconstruction of Aceh and Nias]) (BRR) for a 4-year period until April 2009. In this project, the education sector was embedded in this massive project, either physically reconstructed or rehabilitated emotionally by means of anti-earthquake construction, evacuating buildings and gathering valuable information from those who survived to develop a better strategy for disaster preparedness (BRR 2009). Hence, the spirit of DRR education was embedded into the entire community in Aceh at that time.

Aceh’s disaster has attracted many international actors to help the remaining population and its government’s institutions to recover physically and psychologically. Indonesia had failed and was unable to start working on this situation without international aid and support. While it is possible to retain a sense of humanity and seek more international aid for education, there were also some differences in relation to both responses. Some argued for a stronger sense of national integration and that any response to the disaster should be out of national self-interest. In contrast, the world needed to break down national barriers and an exclusivist notion of DRR, in which case an international response was called for that would see nations cooperating in the name of shared interests in humanitarian works.

Based on the literature, the government became more aware of the importance of disaster education because the catastrophe was a huge blow for it (CDE 2011). The first document published regarding DRR education in Indonesia was the ‘Framework of School-based DRR 2006–2009, World Campaign for Disaster Reduction Integrating Disaster Management at School’. Furthermore, the Consortium for Disaster Education (2011) recognised that: ‘for a country like Indonesia that is exceedingly vulnerable to various threats of disaster, subjects in disaster preparedness have not yet been considered important for schools’ (CDE 2011:5). Even more, this disaster contributed to the collapse of Aceh’s government and the failure of Indonesia to properly respond to this disaster in any sense. Consequently, Indonesia had to open Aceh’s territory to international aid provided by several international agencies: International Non-Government Organisations (INGO), UN institutions and 53 countries were involved, 35 of them from developed countries.^6^ There were about 435 nongovernmental organisations (NGOs), 27 donors and one Indonesian government agency – the Rehabilitation and Reconstruction Bureau (BRR) – that worked to support the province after the catastrophe. This international assistance has been memorialised with the unveiling of a monument in Blang Padang Banda Aceh, Indonesia, inscribed with the words ‘thanks to the world’ (Nazaruddin & Sulaiman 2013:79–80).

Since 2006, the Indonesian society for disaster management (MPBI), together with 22 local and international NGOs and the UN Technical Working Group on DRR, developed two essential activities in Indonesia: a national workshop on ‘building school resilience toward disaster’ on 11 October 2006 and a school road show presented to 16 schools in Jakarta on 12 October 2006 for the purpose of introducing basic disaster preparedness to primary school children (CDE 2011). This evaluation found that despite plenty of enthusiasm among students, teachers and schools’ management, there was insufficient capacity and resources in place for this. This was the initial emergence of the Safe Schools and DRR concept in Indonesia in 2006.

Rahiem et al. (2017), confirmed that:

The tsunami in Aceh is one example of a plethora of aid groups coming from all over the world to help what were primarily Muslim communities, with the majority coming from different social, cultural and religious and non-religious perspectives. (p. 495–514, 500)

The country received a good deal of support from different countries and international organisations during that time. All these activities have helped to strengthen the country’s capacity to respond to emergency situations caused by disasters and also to establish a system that helps to reduce vulnerability, while in turn reducing risks created by disasters in the first place.

## Conclusion

Global education originated in developed states to learn the concerns of other peoples in developing countries and progressively became a global concern through the UN and its affiliations in maintaining peace, security and sustainable development. The landscape can refer to the emergence of DRR education, as initiated by the UN’s humanitarian response to many disasters which occurred globally from 1945 onwards. The accumulation of this lengthy experience (1945–2004) has evidently proved that the knowledge, awareness and skill to respond to disasters had been lacking in developing countries. It was then the beginning of the DRR notion formulated in the UN system by certain experts in 1989. This formulation was subsequently held by UNICEF and became a basic document adopted by LIPI post December 2004 in Indonesia.

Disaster risk reduction education in Indonesia is considered to be external interference that encourages the Indonesian government to establish disaster management and institutions to respond to any potential disasters. Significantly, it is not a completely new subject, as it has experienced many disasters, but because of the lack of dissemination of information and of natural science development, very few people know about DRR knowledge. Furthermore, under the inflexibility of the Dutch colonialist power, this knowledge remained static and yet attracted serious attention from the government until 2004. It emerged from the knowledge on disasters which developed through scientific processes, in terms of how and why a disaster occurs. From this basis, technology was developed to forecast when a disaster may occur and what kind of preparation can be made to prevent or mitigate such disaster risks.

Although progress has been slow, there have been three enabling environments that have helped the transformation of global and DRR education in Indonesia. The Reformation era since 1998, the development of disaster science and the earthquake and tsunami in Aceh have demonstrated Indonesia’s inability to cope with disaster risks and attracted the global community, including the UN and its associated agencies, in addition to INGOs, to participate in emergency, rehabilitation and reconstruction programmes. Within this massive global mobilisation, DRR education found its place to flourish, in which global, national and local collaboration positively occurred. It has contributed to the global commitment of 168 countries to the third priority of the HFA (2005–2014) and the UN Decade for Education for Sustainable Development (2005–2014). These efforts displayed the significant progress of DRR education post 2004, although it was considered to lack the essential basics, that is, disaster science, pedagogic development and human resources. All of these developments need to be integrated, not in an isolated project.

The successful experience of local, national and international DRR initiatives may influence other countries in developing a better DRR curriculum for schools. The development of DRR education would not only depend on either a local community or international initiative but more on an integration and collaboration between them. When international DRR initiatives are not in line with the local situation, it would be a gap, and this article encourages finding out connecting norms between international and national contexts to find a better sustainable DRR education and to fill up the content of global DRR policy in their own context of hazards and vulnerabilities.

## Footnotes

1 On the nature of the UN General Assembly Resolutions, see Kadir (2010).

2 See the UNGA Resolution 2608 (XXIV) on ‘Assistance in Case on Natural Disaster’; 2717 (XXV) 1970:5(b) ‘pre-disaster planning at the national and international level …’).

3 The terminology ‘natural disaster’ used in this article is to adapt with the first terminology used by the UN, as mentioned in the UNGA paper. However, the term ‘natural disaster’ is no longer relevant, because there is nothing natural in disasters. Disasters happen as a result of human input, human vulnerability and social construction (Kelman 2010; Mizutori 2020).

4 The measurement of levels of progress of HFA Priorities for Action: (1) is minor, (2) is relatively small, (3) is not substantial, (4) is substantial, and (5) is a comprehensive achievement.

5 Safe School Leader Countries committed to supporting the implementation of the WISS are: Algeria, Brazil, Costa Rica, Croatia, Ecuador, Finland, Honduras, Indonesia, Iran, Italy, Kyrgyzstan, the Lao People’s Democratic Republic, Lebanon, Mexico, Mongolia, Nepal, Nigeria, Philippines, Saint Vincent and Grenadines, Tunisia, Turkey. The first meeting of Safe School Leaders, with the participation of 16 countries, developed a draft ‘Istanbul Roadmap’, which was presented at the UN World Conference on Disaster Risk Reduction (WCDRR) and will be further revised in close coordination with Safe School Leader Countries, building on the commitments made to WISS and the implementation of safe schools at the Commitments to Safe Schools session (UNISDR 2015b).

6 To memorialise the contributions of 53 countries, Aceh built a monument called the ‘thanks to the world’ monument, which stated ‘thank you’ in the respective languages of these countries.
